# Twelve-Month Outcomes of Anti-VEGF Therapy for nAMD with Brolucizumab, Aflibercept, and Ranibizumab in the Polish National Registry: A Multicenter Database Study

**DOI:** 10.3390/jcm14196771

**Published:** 2025-09-25

**Authors:** Sławomir Teper, Daniel Ledwoń, Patrycja Romaniszyn-Kania, Adam Sendecki, Aleksandra Tuszy, Julia Nycz, Andrzej W. Mitas, Małgorzata Figurska, Edward Wylęgała, Marek Rękas

**Affiliations:** 1 Chair and Clinical Department of Ophthalmology, Faculty of Medical Sciences in Zabrze, Medical University of Silesia, 40-752 Katowice, Poland; slawomir.teper@sum.edu.pl (S.T.); adam.sendecki@gmail.com (A.S.); ewylegala@sum.edu.pl (E.W.); 2 Scientific Research Facility, Branch in Bielsko-Biala, Medical University of Silesia, 40-752 Katowice, Poland; 3 Faculty of Biomedical Engineering, Silesian University of Technology, 41-800 Zabrze, Poland; patrycja.romaniszyn-kania@polsl.pl (P.R.-K.); aleksandra.tuszy@polsl.pl (A.T.); andrzej.mitas@polsl.pl (A.W.M.); 4 Institute of Biomedical Engineering and Informatics, Technische Universität Ilmenau, 98693 Ilmenau, Germany; julia.nycz@tu-ilmenau.de; 5 Department of Ophthalmology, Military Institute of Medicine, Central Clinical Hospital of the Ministry of National Defense, 04-141 Warsaw, Poland; mfigurska@wim.mil.pl (M.F.); mrekas@wim.mil.pl (M.R.); 6 Ministry of Health, 00-952 Warsaw, Poland

**Keywords:** neovascular AMD, anti-VEGF therapy, real-world evidence, national treatment registry, treatment outcomes

## Abstract

**Background/Objectives:** Real-world registries of neovascular age-related macular degeneration (nAMD) treatments provide critical insights for optimizing patient care and resource allocation. This study evaluates one-year outcomes of anti-VEGF therapy with aflibercept, ranibizumab, and brolucizumab in the Polish Therapeutic Program Monitoring System between 1 January 2016 and 31 October 2023. **Methods:** We analyzed data from 51,902 treatment-naïve patients with nAMD, comparing baseline characteristics and outcomes across drugs, as well as between those who discontinued therapy early and those treated for at least one year. **Results:** No significant baseline differences were found between drug groups. One-year follow-up was available for 40,396 eyes; 3184 were lost to follow-up, and 8322 discontinued treatment: 14.4% for those receiving aflibercept, 24.1% for those receiving brolucizumab, and 20.1% for those receiving ranibizumab. Early discontinuers were older and had higher baseline visual acuity (aflibercept, ranibizumab). Twelve-month treatment outcomes, particularly visual acuity gains and injection frequency (~6–7/year), were similar across agents. Only ~22% achieved at least 0.3 logMAR improvement, underscoring real-world treatment challenges. **Conclusions:** System-level support, appropriate treatment intensification, and strategic use of newer, durable agents like brolucizumab are crucial to narrowing the gap between clinical trial efficacy and real-world effectiveness, ultimately improving long-term outcomes in nAMD care.

## 1. Introduction

Age-related macular degeneration (AMD) constitutes an increasingly significant public health concern, driven by aging populations and extended life expectancy [1]. This condition affects individuals over 55 years and jeopardizes the high-resolution central vision essential for critical activities such as reading, driving, and facial recognition. Recent therapeutic developments for neovascular age-related macular degeneration (nAMD) provide the potential for more durable benefits, while ongoing research is making headway towards effective treatments for atrophic forms of late-stage age-related macular degeneration. Nonetheless, a definitive intervention to decelerate disease progression in its early phases or to prevent the onset of advanced AMD has yet to be realized, and our comprehension of the fundamental mechanistic pathways remains in continuous development.

Multiple nationwide registries have provided critical insights into the evolving landscape of nAMD, guiding clinical management and resource allocation in diverse healthcare systems. In Finland, Purola et al. utilized data from three nationwide surveys and registries spanning 2000 to 2017 to delineate the epidemiological trends of nAMD [2]. Their longitudinal analysis demonstrated shifts in incidence and treatment responsiveness correlated with the introduction and widespread use of anti-vascular endothelial growth factor (anti-VEGF) agents, underscoring the utility of comprehensive registries in tracking real-world disease burden and therapeutic impact over extended periods. Similarly, administrative healthcare data from Italy have proven invaluable in characterizing the population burden of nAMD and evaluating anti-VEGF treatment delivery at a national scale. Calabria et al. leveraged these administrative datasets to quantify treatment prevalence and demographic variability, highlighting potential disparities and informing optimization strategies in treatment accessibility and adherence within routine clinical practice [3]. Real-world evidence studies such as the FARIT study by Lupidi et al. further illustrate the role of registries in assessing emerging therapeutics [4]. Their investigation of faricimab, a bispecific antibody targeting both VEGF and Angiopoietin-2, in Italian patients with exudative nAMD delineates both efficacy and safety profiles outside controlled clinical trial settings, contributing to evidence-based expansion of therapeutic options. Systematic reviews and meta-analyses synthesizing data across multiple registries and clinical studies, such as the work by Yen et al. on intravitreal faricimab, provide high-level evidence for therapeutic efficacy and safety, guiding clinical practice guidelines [5]. Additionally, demographic and clinical factors influencing anti-VEGF treatment responses have been systematically reviewed, emphasizing the complex interplay of patient characteristics captured within registry data [6]. Finally, international comparisons using national insurance claims data, including studies from Japan, reveal variations in treatment patterns and healthcare costs, reinforcing the importance of registry data in cross-national health economics evaluations and policy planning [7].

Current treatment modalities predominantly involve repeated intravitreal injections of agents targeting VEGF pathways, with emerging therapies expanding the pharmacologic arsenal [8,9]. Aflibercept, a recombinant fusion protein acting as a decoy receptor for VEGF-A, VEGF-B, and placental growth factor, is widely employed in clinical practice. Recent phase 3 clinical trials, including the PULSAR study, have demonstrated the efficacy and safety of a high-dose (8 mg) aflibercept regimen administered intravitreally over 48 weeks, showing non-inferiority to standard dosing protocols and suggesting potential benefits in extending treatment durability [8]. These findings support dose optimization as a strategy to enhance therapeutic outcomes and reduce treatment burden. Ranibizumab, an early and well-established anti-VEGF monoclonal antibody, remains a cornerstone of nAMD treatment. Its well-documented ability to improve visual function and quality of life has been corroborated in numerous clinical studies and registries globally [10].

Globally, therapeutic strategies for nAMD reflect regional variations in healthcare infrastructure and resource availability. High-income countries benefit from comprehensive treatment protocols and registry data supporting regular and optimized administration of anti-VEGF therapies [11]. Conversely, limited access in resource-constrained settings, as exemplified by Bhutan, underscores an ongoing need to balance efficacy, safety, and feasibility of treatment regimens [12]. In summary, the therapeutic management of neovascular AMD continues to advance through innovations in pharmacologic agents and dosing strategies. Integration of robust clinical trial data with real-world evidence informs personalized treatment approaches aimed at maximizing visual outcomes while minimizing patient burden. Ongoing research and post-marketing surveillance remain essential to refining these strategies and expanding treatment accessibility worldwide.

This study aims to assess the outcomes of the first year of anti-VEGF therapy for nAMD using data from the Retinal Therapeutic Program Monitoring Registry (RTPMR), a part of the Polish Therapeutic Program Monitoring System. In particular, the aim is to compare the treatment outcomes depending on the anti-VEGF agents administered: aflibercept, ranibizumab, and brolucizumab.

## 2. Materials and Methods

### 2.1. Dataset

We used real-world, multicenter data from the RTPMR database covering the period between 1 January 2016 and 31 October 2023. The collected dataset included information on the treatment course of 63,840 eyes of 55,011 patients. It contained detailed information on patient characteristics (age, gender, affected eye, date of diagnosis, reason for treatment discontinuation) and on each control visit, both with and without intravitreal injection, including date, central retinal thickness (CRT), visual acuity (VA), injection status, and drug name, as well as other variables that were not used in this study. The guidelines for the treatment of nAMD within the Polish Therapeutic Program Monitoring System allowed the administration of three anti-VEGF agents: aflibercept, ranibizumab, and brolucizumab (since 1 November 2021). The attending physician made the decision about therapy for each patient at the time of qualification. Depending on the measurement scale, VA was reported using either the Snellen or ETDRS chart. To enable comparison, we converted the values obtained to their logMAR equivalents.

The RTPMR used the following inclusion criteria: (1) presence of active macular neovascularization (MNV) affecting more than 50% of AMD-related lesions; (2) age above 45 years; (3) total degenerative lesion area under 12 optic disc diameters; (4) best-corrected visual acuity (BCVA) ranging from 0.2 to 0.8 on the decimal scale, measured with the Snellen chart or its ETDRS equivalent (34–80 letters); (5) patient agreement to undergo intravitreal injections; and (6) no dominant geographic atrophy, hemorrhage, or significant irreversible foveal damage, including fibrosis, foveal atrophy, or substantial chronic scarring. The decision regarding a patient’s eligibility for treatment was made and verified by independent members of the Coordination Committee based on an application submitted to the system by the attending physician.

In this study, we excluded eyes that had received prior treatment outside the Polish Therapeutic Program Monitoring System, retaining only treatment-naïve subjects. During the analysis, we identified eyes that were lost to follow-up because of too short current observation time or treatment discontinuation before completing the first year of therapy. We compared the patients’ characteristics and outcome measures between eyes that terminated treatment earlier and the rest, treatment-naïve, with at least one year of follow-up. Then, we evaluated the treatment outcomes of twelve-month anti-VEGF therapy depending on the drug administered.

The RTPMR data are anonymized and de-identified, so institutional review board approval and written informed consent were not necessary for this study. The research was conducted in accordance with the principles of the Declaration of Helsinki.

### 2.2. Outcome Measures

We analyzed the characteristics of the study group, focusing on the relationships between the initial anti-VEGF drugs administered and all patients’ sex, age, VA, and CRT. For the treatment discontinuation group, we evaluated the proportions of eyes with different reasons of treatment discontinuation reported in RTPMR and compared the number of injections and time to termination depending on the drug administered. Then, we compared sex, age, VA (initial and after first injection), and CRT (initial and after first injection) between the treatment discontinuation and 12-month therapy groups. For the one-year follow-up group, we evaluated the final VA and CRT and their relative changes depending on anti-VEGF therapy using exploratory analysis and univariate and multivariate statistical analysis.

### 2.3. Statistical Analysis

We used the Kruskal–Wallis test with epsilon squared ($\epsilon^{2}$) as the effect size measure, followed by Dunn’s post-hoc test with Bonferroni correction to compare quantitative variables between three groups corresponding to administered anti-VEGF agents. The two groups’ comparisons between discontinued treatment and one year of follow-up were made with an independent *t*-test or Mann–Whitney U test, depending on whether the normality (Shapiro–Wilk test) and homogeneity of variances (Levene’s test) assumptions were met. We measured the effect size using Cohen’s d, or rank-biserial correlation (rg), respectively. The proportions of categorical variables between groups were compared using the chi-square test, with effect size reported as Cramér’s V.

In multivariate analysis, a multinomial logistic regression model via neural networks was used to predict the probability of selecting a specific drug, depending on the initial VA, initial CRT, age, and gender of the subjects. Generalized linear models with a Gaussian function of error distribution without and with interaction between anti-VEGF agent and age were used to model final VA depending on the drug administered, the number of injections per year, sex, and age of the patient.

## 3. Results

### 3.1. Study Group Characteristics

We found no significant relationships between the initial anti-VEGF agents administered and the age, VA, and CRT of all patients in the RTPMR. The mean baseline VA for each drug was (logMAR): aflibercept 0.47 ± 0.24, ranibizumab 0.46 ± 0.23, brolucizumab 0.43 ± 0.21 (*p* < 0.001, $\epsilon^{2}$ = 0.0013—very small effect size). The analyzed dataset contained 51,902 treatment-naïve eyes among all 63,840 eyes in RTPMR. The 38,240 eyes started treatment with aflibercept, 12,432 with ranibizumab, and 1230 with brolucizumab. In the treatment-naïve group, data from at least one year of treatment were collected for 40,396 eyes, while the remaining 11,506 were lost to follow-up for the following reasons: treatment of 3184 eyes lasted less than one year at the time of data collection, and 8322 (16.0% of all treatment-naïve eyes) terminated treatment before the end of the first year of therapy.

### 3.2. Reasons for Discontinuing Treatment During the First Year of Therapy

**Figure 1:**
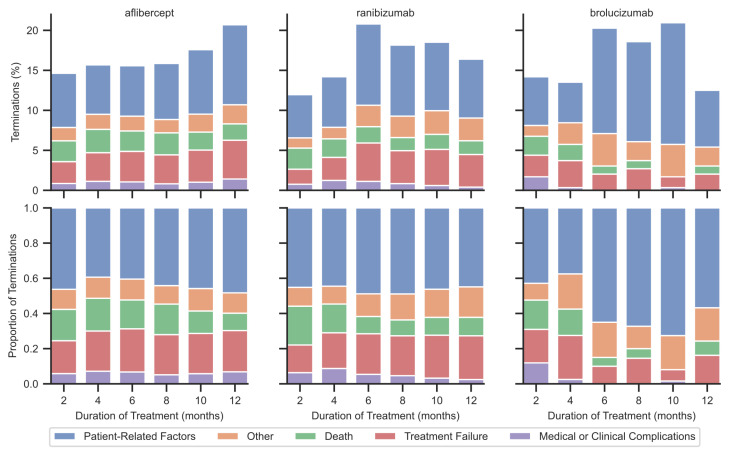
Discontinuation rates and the relative contribution of each reason for treatment discontinuation over time, stratified by anti-VEGF agent.

In the first year of therapy, the proportions of treatment discontinuation among treatment-naïve patients were 14.4% for those receiving aflibercept (n = 5525), 24.1% for those receiving brolucizumab (n = 296), and 20.1% for those receiving ranibizumab (n = 2501). These differences are statistically significant, but with a very small effect size (chi-square, *p* < 0.001, Cramer’s V = 0.074). The number of injections at the end of therapy differed significantly between drugs (Kruskal–Wallis test, *p* < 0.001, $\epsilon^{2}$ = 0.019). Pairwise comparison in the post-hoc test showed a significant difference between aflibercept and the other drugs (Dunn’s test: aflibercept vs. brolucizumab and aflibercept vs. ranibizumab, *p* < 0.001): aflibercept 4.55 ± 1.94, ranibizumab 4.03 ± 1.94, brolucizumab 4.01 ± 1.52 injections. At the same time, no significant differences were found in the time to treatment discontinuation depending on the drug administered (*p* = 0.081). The average time to discontinuation in the first year of treatment was 198.6 ± 106.0 days. The numbers and proportions of the individual causes of all treatment discontinuation, stratified by drug, are presented in Table 1. For aflibercept, discontinuation increased at the end of the treatment year, while for ranibizumab and brolucizumab it peaked at month six (Figure 1). The general reasons for termination over time were comparable for aflibercept and ranibizumab. For brolucizumab, patient-related and other factors predominate from the sixth month.

Early treatment discontinuation (before receiving three injections of the drug) was most common for ranibizumab (18.9% of discontinuations among those treated with ranibizumab). For brolucizumab, this percentage was 13.9% and for aflibercept, 15.7%. Regardless of the drug, half of all early discontinuations occurred within the first 2 months of treatment, and at least 90% within the first 6 months. The distribution of causes of early termination is similar to the distribution of causes of all terminations—patient-related factors, death, and other factors predominate.

### 3.3. Comparison Between Eyes with Treatment Discontinuation and with at Least One Year of Follow-Up

Comparisons of the characteristics of the discontinued group with the group treated for at least 12 months, stratified by drug, are presented in Table 2.

For each drug, the group that discontinued treatment earlier was significantly older at the start of treatment than the group that continued treatment. The proportions of males and females did not differ between groups for any anti-VEGF agent. The group that discontinued treatment earlier started treatment with significantly higher VA for aflibercept and ranibizumab. This relationship persisted in VA measurements after the first injection, despite a decrease in mean VA in both groups. For brolucizumab, the difference in VA between the discontinuing and continuing groups was significantly smaller. No significant differences were found between groups for either drug in CRT measurements. However, in the case of brolucizumab, a lower CRT value was found after the first injection in the group discontinuing treatment compared to the continuing group.

### 3.4. Year of Treatment Outcomes

**Figure 2:**
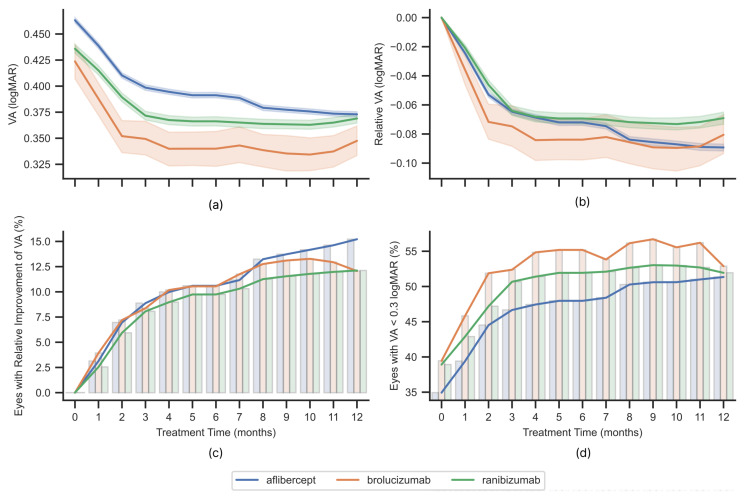
The visual acuity (VA) outcomes during first twelve months of anti-VEGF treatment with different drugs: mean with confidence intervals (**a**,**b**), percentage of eyes with improvement (**c**), and percentage of eyes with VA < 0.3 logMAR (**d**). Relative measures (**b**,**c**) refer to the values during qualification to the treatment.

The mean VA values (with confidence intervals for the mean) in subsequent months of therapy are presented in Figure 2a. For months with missing data, the last measurement obtained was duplicated.

The plot of VA changes relative to the measurement at the time of qualification is presented in Figure 2b. Brolucizumab is characterized by earlier achievement of greater improvement, particularly in comparison with ranibizumab, for which the average acuity at the time of inclusion was at a similar level. The upper limit of the confidence interval in subsequent months coincides with the improvement achieved with ranibizumab. Up to 6 months, the average change in visual acuity follows a similar pattern for aflibercept and ranibizumab. In the case of aflibercept, there was a greater average improvement in visual acuity after this period, and the decreasing trend in VA (logMAR) continues up to 12 months of treatment.

Figure 2c shows the percentage of patients who achieved an improvement in visual acuity of at least 0.3 logMAR compared to the value at the time of qualification in subsequent months of treatment. Figure 2d shows the percentage of patients achieving VA < 0.3 logMAR in subsequent months of treatment.

The VA after one year of therapy does not differ significantly depending on the drug administered (Kruskal–Wallis: *p* = 0.0001, $\epsilon^{2}$ = 0.0004). Similarly, no relationship was found between the change in VA relative to the measurement at the time of qualification and the drug taken (Kruskal–Wallis: *p* < 0.001, $\epsilon^{2}$ = 0.002). At least one improvement in visual acuity of at least 0.3 logMAR compared to the initial measurement was achieved by 23.1% of patients receiving aflibercept, 21.9% receiving brolucizumab, and 21.5% receiving ranibizumab. Among patients starting treatment with VA > 0.3 logMAR, at least once in the first year of therapy, 51% achieved VA < 0.3 logMAR with aflibercept, 57.3% with brolucizumab, and 55.4% with ranibizumab.

### 3.5. Multivariate Analysis

The results of the multinomial logistic regression model for predicting the probability of selecting a specific drug based on patient characteristics at the time of qualification are presented in Table 3. Worse baseline VA significantly reduces the likelihood of choosing both brolucizumab and ranibizumab, which may indicate a preference for aflibercept in patients with more advanced visual impairment. Retinal thickness has a very small effect on treatment choice. These changes are statistically significant, but their effect size is minimal. Sex and age do not influence drug choice.

The final VA regression model results showed an increase of 0.004718 logMAR (0.24 ETDRS letters) for each year of life in patients treated with aflibercept (the older the patient, the worse the result). For patients treated with brolucizumab, initial age also worsens the result, but to a lesser extent than with aflibercept (an increase of 0.003 logMAR (0.15 ETDRS letters). Patients treated with ranibizumab show the weakest effect of age on vision deterioration (Table 4). It means that older patients may achieve slightly better final results when using alternative drugs to aflibercept.

The linear regression model, with adjustment for baseline status, showed that the final VA after one year of treatment depends primarily on baseline VA (a strong predictor—each 0.1 logMAR decrease in visual acuity at baseline is associated with a 0.07 logMAR decrease in visual acuity at the end of treatment) (Table 5). Age is also a statistically significant predictor of final VA (for each additional year of the patient’s life, the final VA increases by 0.0013 logMAR). Males have worse final results than females, considering the initial condition, by 0.0028 logMAR, and the number of injections performed per year is associated with a slight improvement in final VA (by 0.0010 logMAR). Ranibizumab was associated with a slightly worse effect compared to aflibercept (by 0.0084 logMAR), while the differences between brolucizumab and aflibercept were not statistically significant.

## 4. Discussion

### 4.1. Comparison with Global Clinical Trials and Real-World Studies

The 12-month outcomes in our national registry can be contextualized against pivotal trials and large cohort studies. In landmark RCTs like MARINA and ANCHOR, monthly ranibizumab demonstrated substantial vision benefits: ~95% of treated eyes avoided moderate vision loss and ~33–40% achieved ≥15-letter gains at 1 year [13,14]. Mean VA improvements of +7 to +11 letters were reported with monthly ranibizumab in these trials. Similarly, the VIEW 1 and 2 trials showed that fixed regimens of ranibizumab or aflibercept can yield ~+8 letter gains at 52 weeks, with ~30% of patients gaining ≥15 letters [15]. By contrast, our real-world cohort’s VA gains (modestly positive on average) fall short of these RCT benchmarks—a common observation globally. Real-world registries typically report smaller VA gains or stabilization, attributed to less intensive treatment. For example, the IRIS Registry in the United States found an overall mean change of approximately 0 to +3 letters at 1 year, markedly lower than RCT outcomes, largely due to under-treatment in routine practice [16]. In our study, only ~22% of eyes attained ≥15-letter gains (approximately one in five), which is lower than the one-third of patients achieving such gains in tightly controlled trials. This discrepancy aligns with other real-world data (e.g., the multi-country AURA study and UK EMR audits) showing that the stringent efficacy of trials is difficult to replicate in clinic settings, primarily because patients receive fewer injections and have more heterogeneous follow-up [17,18].

Notably, our cohort’s injection frequency was far lower than the ≥12 injections delivered in monthly-dosing trials, which likely contributed to the more modest vision gains. In modern practice, however, strict monthly dosing has largely been supplanted by treat-and-extend (T&E) regimens. T&E allows individualized extension of treatment intervals to reduce injections and clinic visits while maintaining efficacy, and it has been widely adopted globally in nAMD management. A systematic review confirmed that T&E yields visual outcomes comparable to fixed monthly dosing over 1–2 years with significantly fewer injections [19]. Moreover, patients on T&E achieve better VA gains than those on reactive PRN schedules, by avoiding undertreatment [19]. Consistent with this, an IRIS registry analysis of 184,000+ eyes showed that >40% of patients received <7 injections in the first year, and those under-treated eyes had essentially no vision improvement (mean ~0-letter change), whereas eyes receiving ≥7 injections had modest positive outcomes (~+3–4 letters) [20]. This underscores the impact of treatment intensity on outcomes. Real-world studies from other countries reinforce this pattern: the Fight Retinal Blindness! (FRB) registry (Australia) reported +3 to +5 letter gains with pro re nata or T&E regimens over 12 months, achieved with a mean of ~8 injections [21,22].

Some registry studies have directly compared anti-VEGF agents’ performance outside of trials. Our finding that all three agents—aflibercept, ranibizumab, and brolucizumab—yielded comparable 1-year vision outcomes is in line with prior real-world reports. For instance, Gillies et al. found no significant VA difference between eyes treated with ranibizumab vs. aflibercept in routine practice (both groups gaining ~+4 letters) when baseline covariates were matched [21]. An updated FRB analysis by Bhandari et al. noted a slightly higher mean 1-year gain in aflibercept-treated eyes than ranibizumab-treated eyes (+5.0 vs. +2.9 letters) in an unmatched cohort [22], but such differences were modest. Our registry’s inclusion of brolucizumab provides one of the first large-scale real-world comparisons involving this newer agent. In the pivotal HAWK and HARRIER trials, brolucizumab (dosed every 12 or 8 weeks after loading) was non-inferior to aflibercept (every 8 weeks) in year one, with ~+6.6 to +6.9 letters gained at 48 weeks—similar to aflibercept’s ~+6.8 to +7.6 letters [23]. Those trials showed ~25–33% of patients on brolucizumab achieved ≥15-letter gains (comparable to aflibercept). Notably, approximately half of brolucizumab-treated eyes in HAWK/HARRIER were able to be maintained on a 12-week dosing interval through the first year without disease reactivation—reflecting the drug’s longer durability of effect [23]. This durability advantage is attributed to brolucizumab’s high molar concentration and smaller molecular size, allowing extended VEGF suppression. Emerging real-world evidence further corroborates brolucizumab’s potential for extended efficacy. In a large Japanese registry study, 62.3% of treatment-naïve brolucizumab eyes were free of retinal fluid at 12 months (vs. ~10% at baseline), demonstrating robust disease control [24]. In pre-treated eyes from the same study, the median injection interval was extended by 21 days by month 12 (relative to baseline), indicating that many patients could be managed on longer intervals [24]. Furthermore, a 2-year prospective T&E study reported that 47% of eyes on brolucizumab met criteria to discontinue treatment after achieving two consecutive 16-week intervals, with only ~5–6% experiencing a relapse in the year following cessation [25]. These data highlight that, in select patients, brolucizumab can maintain long-term disease quiescence and substantially reduce injection frequency. In our real-world data, we likewise observed no significant difference in 12-month VA gains between brolucizumab and aflibercept (or ranibizumab)—any slight advantage in early anatomical drying with brolucizumab did not translate into meaningfully better vision by 1 year, likely due to individualized dosing and the influence of other factors in practice.

Overall, the comparative effectiveness of anti-VEGF agents in our national cohort aligns with global experience: treatment intensity and baseline lesion characteristics appear to drive outcomes more than the choice of agent when using any approved anti-VEGF therapy. Our findings bolster the consensus that achieving trial-like outcomes in nAMD requires maximizing injection frequency and adherence in real-world settings. Variations from country to country (and versus trials) further emphasize the need to close the gap between protocol-driven therapy and actual practice.

### 4.2. Clinical and Health System-Specific Distinctions of Our Study

The Polish Retinal Therapeutic Program has specific inclusion criteria (e.g., a defined range of baseline visual acuity and lesion characteristics) that ensured a relatively broad yet standardized enrollment of treatment-naïve nAMD eyes. Notably, extremely low-vision eyes with end-stage fibrosis or atrophy are generally not started on anti-VEGF in the program, which differs from U.S. real-world cohorts (like IRIS) that may include such cases regardless of baseline vision. This partly explains why our registry’s baseline VA (~20/60 on average) was slightly better than in some U.S. studies. It suggests that in Poland, patients are referred and treated before vision deteriorates to very low levels. By contrast, analyses from the U.S. have found a substantial subset (≈15%) of nAMD patients begin treatment at ~20/200 or worse, which portends a poorer prognosis since eyes starting with very low vision rarely improve to functional levels [16]. Our program’s practice of not initiating therapy in eyes with irreversible damage (disciform scars or advanced atrophy) means our outcomes are not diluted by eyes essentially destined for limited response. This “ceiling effect” of baseline VA is well recognized in real-world AMD outcomes—eyes presenting above 20/40 can often maintain good vision, whereas those starting very low have limited potential for gain.

A key system-specific factor is the structured reimbursement and monitoring regimen in the Polish Therapeutic Program. All patients received three initial loading doses and then typically continued on a pro re nata regimen that was changed around 2020 to a treat and extend schedule with regular visits. However, constraints on visit frequency and resource limitations can influence the achieved injection counts. The median injection count is comparable to other middle-income European settings; for instance, the AURA multi-country study reported a mean of ~5 injections in the first year in routine care, with significant country-to-country variation [17]. The 12-month outcomes in our cohort mirror those in countries that employ PRN dosing with similar injection numbers. One distinction is that treat-and-extend protocols have been increasingly adopted elsewhere to improve efficiency and outcomes; however, in our national database period (2019–2021), treat-and-extend was not yet universally implemented, as many practitioners followed a conservative monthly-monitoring PRN approach under program guidelines. The recent Italian “BIVIR” study, which instituted a lean-management treat-and-extend clinic model, achieved a mean of 6.1 injections in Year 1 (very close to our mean) yet reported a +6.3-letter visual gain—higher than our ~+3 to +5-letter gain in Year 1 [26]. This difference may reflect more aggressive visit scheduling or demographic differences, despite similar injection counts. It underscores that not just the number of injections but also how they are timed (continuous treat-and-extend vs. PRN gaps) can affect outcomes.

The overall 12-month dropout rate in our cohort was 16.0%, which is relatively low compared to some reports (for example, 22–25% one-year loss to follow-up in certain U.S. clinic series). This can be attributed to the structure of our Therapeutic Program—treatment is delivered in specialized centers with appointment tracking mandated by the national funding system, which reduces unmonitored attrition. Moreover, full financial coverage of anti-VEGF drugs by the public payer removes the cost barrier that often contributes to dropouts in other health systems. In contrast, analyses from predominantly private-pay systems (e.g., the U.S.) indicate that economic and access issues contribute to higher early discontinuation rates.

The significantly higher rate of discontinuations because of “no active treatment within 4 months” suggests that some physicians attempted to extend brolucizumab dosing intervals or temporarily paused treatment due to perceived disease quiescence, given its longer durability. This nuance is specific to our program’s protocol, which defines prolonged inactivity as discontinuation for audit purposes. In effect, brolucizumab’s very strength (maintaining disease control for longer intervals) paradoxically led to administrative “dropouts” in our dataset. Notably, these instances likely represent eyes that were doing well and did not truly exit care, underscoring brolucizumab’s potential to reduce treatment frequency (similar long-term remissions on brolucizumab have been reported in treat-and-extend-and-stop studies [25]).

Additionally, patient-driven discontinuations (withdrawal of consent) were more common with aflibercept than with brolucizumab. This may reflect that brolucizumab was often used in more motivated patients or those under closer follow-up due to its new status, whereas aflibercept (being introduced earlier) had a broader user base, including some less adherent patients. Overall, the structured nature of our national registry and treatment program likely improved adherence relative to more fragmented systems, yet it also imposed strict definitions (like the 4-month rule) that influence reported outcomes. It should be emphasized, however, that returning to the program is easy, and we also analyzed patients for return to treatment after completing their previous program. The difference between patient follow-up during and after the program is limited only to the discretion of the visit intervals if they are outside the program. In the first year, this phenomenon is very limited in significance.

### 4.3. Strengths of the Study

Our study’s strengths lie in its unprecedented scale, real-world representativeness, and the granularity of data from a national registry. First, with over 40,000 treatment-naïve eyes and a standardized 12-month follow-up, this analysis is among the largest nAMD outcome studies globally, second only to the AAO’s IRIS registry in sample size (for comparison, the IRIS registry analysis included >100,000 eyes [16]). Such a large cohort provides high statistical power to detect even small differences in outcomes and allows robust subgroup analyses that smaller studies could not reliably perform.

The data are drawn from a nationally implemented therapeutic program, meaning our findings reflect real-world practice across numerous centers. This enhances generalizability: the outcomes encompass a diversity of practitioners and patient demographics, mirroring “true” population-level effectiveness of anti-VEGF therapy in a public healthcare setting.

The RTPMR design ensured prospective data capture and near-complete documentation. Every anti-VEGF injection and visit was logged as part of reimbursement requirements, minimizing selection bias and missing data. We had access to reasons for discontinuation, injection counts, and outcomes for virtually all patients who met inclusion criteria—a notable strength over retrospective chart reviews that often suffer from incomplete follow-up.

The inclusion of three major anti-VEGF agents in the same registry under uniform conditions enabled a contemporaneous comparison of their performance. This head-to-head real-world insight is unique—especially for brolucizumab, which had limited real-world data at the time. Our study, therefore, provides valuable early evidence on brolucizumab’s routine use in a large cohort, including its efficacy, durability, and safety profile relative to established therapies. Finally, the study collected both functional (VA) and anatomical (CRT) outcomes. This allows correlation of vision gains with disease activity resolution, strengthening our conclusions. The breadth of data on anatomical response and safety (e.g., incidence of adverse events leading to discontinuation) adds a comprehensive perspective that reinforces the reliability of our findings. In summary, the scale, completeness, and real-world nature of this registry-based study ensure that the conclusions drawn are robust and relevant to everyday clinical practice and health policy planning.

### 4.4. Limitations

In our real-world observational research, treatment was not randomly assigned to aflibercept, ranibizumab, or brolucizumab; rather, ophthalmologists chose the agent based on clinical judgment, drug availability, or timing. Although baseline VA and CRT were statistically similar across the three groups in our cohort, unmeasured confounders may have influenced which patients received a particular drug. We attempted to mitigate bias by analyzing outcomes primarily within each agent and adjusting for baseline vision in regression models, but residual confounding cannot be eliminated.

Our registry did not mandate a fixed treatment regimen after loading doses—some patients were managed PRN, others on treat-and-extend at the physician’s discretion. This heterogeneity means the “exposure” to treatment differed between patients, and outcomes represent an average of various dosing patterns. Eyes that discontinued early were included in the analysis using last observation carried forward, which might underestimate the benefits in patients who would have continued therapy longer. Conversely, our outcomes could be inflated if physicians stopped treatment in eyes doing very poorly (attrition bias), as those eyes’ later decline would not be captured.

Visual acuity measurements were obtained in routine clinics and might have variability. OCT machines were not uniform, and CRT measurements from different devices were pooled, possibly adding noise to anatomical outcomes. However, given the large sample, such inter-center variability likely averages out and would only marginally affect aggregate results.

The smaller sample size of brolucizumab in our study and the fact that it was available only in the latter part of the study window mean this cohort had slightly shorter average follow-up time and less power to detect rare outcomes. The incidence of brolucizumab-related adverse events recorded in our data was low, but this almost certainly underrepresents the true rate of immune-mediated side effects. Some inflammatory events might not have been explicitly coded or may have occurred just after the 12-month cutoff. Furthermore, heightened vigilance in early post-marketing might have led to under-reporting of mild cases. Thus, conclusions on brolucizumab’s safety signals from our study should be drawn carefully, and our analysis was not primarily designed to assess safety endpoints.

### 4.5. Future Directions and Clinical Recommendations

A clear message from our data and global comparisons is that real-world patients often receive suboptimal injection frequency, which curtails visual gains. Efforts should be directed at closing the gap between trial-proven regimens and actual practice. Embracing treat-and-extend protocols more broadly could reduce overall clinic visits while maintaining a higher frequency of treatment, as evidenced by reports that treat-and-extend approaches yield better long-term vision retention than reactive PRN dosing in many settings [19].

Additionally, our finding that a large number of discontinuations were triggered by perceived inactivity or patient choice suggests that improved counseling about the chronic, progressive nature of nAMD could enhance adherence. Implementing reminder systems or dedicated follow-up coordinators might also prevent no-show appointments and inadvertent gaps in treatment — for instance, avoiding intervals beyond the 4-month threshold that in our program automatically led to discontinuation classification. Currently, due to the emergence of drugs with a prolonged duration of action, this period has been extended to 5 and soon 6 months.

Our data hinted that certain subgroups respond differently to treatment intensity and may require tailored approaches. Moving forward, personalized treatment algorithms could be developed. The RTPMR dataset will allow future analyses into early predictors of long-term non-response, which can inform such decision-making. Moreover, the unique discontinuation patterns observed with brolucizumab suggest that this agent offers an extended durability that could be harnessed beneficially [25]. The fact that many brolucizumab-treated eyes went >4 months dry underscores its potential to reduce treatment burden in select patients. On the other side, the intraocular inflammation rates on the order of 3–4% in brolucizumab-treated eyes [27], reaffirming that while such events are uncommon, they are higher than with prior agents and necessitate vigilance. Our program will continue to monitor safety outcomes in routine use.

Finally, our findings underscore the need for system-level support to achieve optimal outcomes in nAMD management. Reducing dropout rates and ensuring continuous care will require interventions beyond what individual clinics can do. For example, involving primary ophthalmologists or referring doctors in ongoing patient education about the importance of follow-up, providing transportation or home-visit services for patients with mobility issues (to mitigate non-medical barriers to care), and leveraging telemedicine for interim check-ins could all help prevent avoidable loss to follow-up. Health authorities re-evaluated the rigid 4-month inactivity rule: while it ensures accountability and discourages lax follow-up, a slightly longer allowable interval (e.g., 5–6 months in select stable cases) prevents classifying still-engaged patients as “dropouts” when in fact the physician’s intent was to extend the treatment interval. Any such policy changes need to be balanced with patient safety.

In conclusion, the first-year results from the Polish national nAMD registry not only reflect treatment effectiveness to date but also chart a course forward. By intensifying treatment when needed, leveraging new therapeutics like brolucizumab (and newer agents) for their durability, and refining program policies, we aim to better translate the promising outcomes seen in clinical trials into routine practice—thereby improving long-term visual prognosis for our aging population.

## Figures and Tables

**Table 1:** Numbers and proportions of eyes with treatment discontinuation for specific reasons in the Polish Therapeutic Program Monitoring System, stratified by anti-VEGF agent.

Reason for Treatment Discontinuation	Aflibercept	Ranibizumab	Brolucizumab
Patient-Related Factors			
No active treatment within 4 months	1042 (18.9%)	671 (26.8%)	153 (51.7%)
Patient’s withdrawal from treatment	1023 (18.5%)	317 (12.7%)	13 (4.4%)
Lack of cooperation between the patient and the attending physician	382 (6.9%)	179 (7.2%)	9 (3.0%)
Other	647 (11.7%)	347 (13.9%)	50 (16.9%)
Death	835 (15.1%)	306 (12.2%)	22 (7.4%)
Treatment Failure			
Disease progression	636 (11.6%)	337 (13.5%)	14 (4.9%)
Presence of permanent damage to the fovea structure			
Other permanent lesions	186 (3.4%)	78 (3.1%)	9 (3.0%)
Fibrosis	213 (3.9%)	64 (2.6%)	9 (3.0%)
Atrophy	103 (1.9%)	43 (1.7%)	2 (0.7%)
Significant chronic disciform scar	112 (2.0%)	35 (1.4%)	8 (2.7%)
Medical or Clinical Complications			
Adverse reactions associated with the active substance	227 (4.1%)	85 (3.4%)	3 (1.0%)
Hypersensitivity to the active substance	26 (0.5%)	20 (0.8%)	3 (1.0%)
Tractional retinal detachment or a full thickness macular hole	40 (0.7%)	5 (0.2%)	0 (0.0%)
Active severe endophthalmitis	36 (0.7%)	6 (0.2%)	0 (0.0%)
Active infection of the eye or its vicinity	17 (0.3%)	8 (0.3%)	1 (0.3%)

**Table 2:** Patient and treatment characteristics of eyes with treatment discontinuation and those with at least one year of follow-up, stratified by drug.

	Aflibercept			Ranibizumab			Brolucizumab		
	12-Months Treatment (n = 30,226)	TreatmentDiscontinued(n = 5525)	*p*-Value, Effect Size	12-Months Treatment (n = 9559)	TreatmentDiscontinued(n = 2501)	*p*-Value, Effect Size	12-Months Treatment (n = 611)	TreatmentDiscontinued(n = 296)	*p*-Value, Effect Size
Age (years)	75.30 ± 8.08	78.42 ± 8.07	<0.001, d = −0.38	75.36 ± 8.31	78.87 ± 8.23	<0.001, d = −0.42	74.88 ± 7.96	76.86 ± 8.69	<0.001, d = −0.23
Sex			<0.001 V = 0.023			0.052			0.31
Female	19,113	3321		6021	1522		402	184	
Male	11,113	2204		3538	979		209	112	
VA (logMAR)									
Initial	0.46 ± 0.23	0.57 ± 0.24	<0.001, rg = 0.25	0.44 ± 0.21	0.54 ± 0.24	<0.001, rg = 0.24	0.42 ± 0.21	0.46 ± 0.23	0.011, rg = 0.10
After first inj.	0.42 ± 0.22	0.55 ± 0.25	<0.001, rg = 0.30	0.40 ± 0.22	0.52 ± 0.25	<0.001, rg = 0.28	0.37 ± 0.21	0.42 ± 0.24	0.005, rg = 0.12
CRT (μm)									
Initial	355 ± 147	380 ± 160	<0.001, rg = 0.08	359 ± 141	373 ± 163	<0.001, rg = 0.07	346 ± 111	357 ± 144	0.61
After first inj.	279 ± 111	290 ± 136	<0.001, rg = 0.02	292 ± 104	302 ± 121	0.12	276 ± 83	262 ± 77	0.008, rg = −0.11

**Table 3:** Parameters of the multinomial logistic regression model, with Afilbercept as the reference category of drug.

	Brolucizumab				Ranibizumab			
	z-Value	*p*-Value	Coefficient	SE	z-Value	*p*-Value	Coefficient	SE
intercept	19.32	<0.001	1.179	0.061	−41.61	<0.001	−1.920	0.046
Initial VA	−26.85	<0.001	−0.824	0.031	−10.95	<0.001	−0.228	0.000
Initial CRT	1.13	0.12	0.000	0.000	−7.16	0.083	−0.001	0.021
Age	−51.25	0.078	−0.042	0.001	16.59	0.058	0.010	0.001
Sex	2.25	0.024	0.031	0.014	0.34	0.73	0.003	0.010

**Table 4:** Results of generalized linear models for final VA, without interaction between anti-VEGF agent and age.

	t-Value	*p*-Value	Coefficient	SE
intercept	9.77	<0.001	0.0451	0.0046
Brolucizumab	−17.95	<0.001	−0.0269	0.0015
Ranibizumab	3.40	<0.001	0.0036	0.0010
Injection count	−3.16	0.0016	−0.0009	0.0003
Sex	9.83	<0.001	9.8310	0.0008
age	88.02	<0.001	0.0044	0.0001

**Table 5:** Results of generalized linear models for final VA, with interaction between anti-VEGF agent and age.

	t-Value	*p*-Value	Coefficient	SE
intercept	−4.04	0.001	−0.0125	0.0031
Initial VA	618.67	<0.001	0.7372	0.0012
Brolucizumab	−1.80	0.071	−0.0018	0.0010
Ranibizumab	11.85	<0.001	0.0084	0.0007
Injection count	−4.56	<0.001	−0.0010	0.0002
Gender	4.92	<0.001	0.0028	0.0006
Age	38.45	<0.001	0.0013	0.0000

## Data Availability

The datasets generated and analyzed during the current study are available from the corresponding author on reasonable requests.
